# Time to meet energy requirements in enteral nutrition and its impact on patient tolerance and clinical outcomes in the ICU

**DOI:** 10.1186/cc10209

**Published:** 2011-06-22

**Authors:** FVCD Marco, JM Souza, C Harmbacher, ALG Guimares

**Affiliations:** 1 Hospital Vivalle, São Jose Dos Campos - SP, Brazil

## Introduction

Delivering early nutrition support therapy, primarily using the enteral route, is seen as a strategy that may reduce disease severity, diminish complications, decrease length of stay in the ICU, and favorably impact patient outcome. SCCM and ASPEN guidelines support that after the initiation of enteral feeding we have 10 days to meet 100% of predicted energy requirements before we consider supplementation with parenteral nutrition (PN). There are scarce data about the clinical effects of using a more accelerated approach to reach full caloric adequacy with enteral nutrition (EN).

## Objective

The aim of this observational study is to evaluate whether a diminished time to target caloric goal is associated with more patient intolerance and clinical benefits in ICU patients receiving EN.

## Methods

From January 2010 to June 2010 we prospectively followed all consecutive ICU patients receiving EN. We collected epidemiological data, APACHE II score, LOS (ICU and hospital), need for mechanical ventilation, incidence of nosocomial infection and hospital mortality. We also collected data on nutrition therapy as the time to target caloric goal (120 hours), total time on nutrition therapy, incidence of diarrhea and other signs of EN intolerance (vomits, abdominal pain and distension). For statistical analysis we used the Kolmogorov-Smirnov test, Student's *t *test and Pearson's correlation coefficient.

## Results

We enrolled 32 patients (17 male/15 female) in the study. The mean age was 66 ± 18 years, mean APACHE II score 21 ± 9, mean ICU and hospital LOS were 21.3 and 35 days respectively, incidence of nosocomial infection was 21.8%, mean total time in nutrition therapy was 18.3 ± 14 days and hospital mortality was 28%. There was need for mechanical ventilation in 56%. There was need for PN supplementation in 9.4% (*n *= 3) of patients. Comparing the different groups (120 hours, *n *= 16) we were unable to detect any difference with statistical significance regarding incidence of diarrhea, EN intolerance, need for MV, total time on nutrition therapy, incidence of nosocomial infection, ICU and hospital LOS and hospital mortality.

## Conclusion

These preliminary data have shown no correlation of a diminished time to meet energy requirements in EN with patient tolerance to nutrition therapy and clinical benefits.

